# Smartphone and Paper-Based Fluorescence Reader: A Do It Yourself Approach

**DOI:** 10.3390/bios10060060

**Published:** 2020-06-02

**Authors:** Laura Alejandra Ireta-Muñoz, Eden Morales-Narváez

**Affiliations:** Biophotonic Nanosensors Laboratory, Centro de Investigaciones en Óptica A. C., León 37150, Mexico; laura.iret@gmail.com

**Keywords:** biosensing, point-of-care, photoluminescence, portable devices, miniaturization

## Abstract

Given their photoluminescent character, portable quantum dot readers are often sophisticated and relatively expensive. In response, we engineered a “do it yourself” fluorescence reader employing paper materials and a mid-range smartphone camera. Black paperboard facilitated a versatile, lightweight and foldable case; whereas cellophane paper was observed to behave as a simple, yet effective, optical bandpass filter leading to an advantageous device for the quantitative interrogation of quantum dot nanocrystals concentrations (from 2.5 to 20 nM), which are suitable for optical point-of-care biosensing. The streptavidin-coated nanocrystals employed are commercially available and the developed reader was benchmarked with a standard portable quantum dot reader, thereby demonstrating advantages in terms of cost and linear analytical range.

## 1. Introduction

Paper is a versatile material to work with; it is relatively cheap and easy to handle given its lightweight and flexible character. In this regard, paper-based analytical devices are amenable to simple and effective on-site testing in different applications, including diagnostics, environmental monitoring and food analysis [1,2,3,4,5]. Moreover, paper-based analytical devices are also amenable to the integration with portable technologies such as smartphones and drones [4,6]. 

Highly sensitive point-of-care biosensing can be critical to enable timely healthcare decisions [7,8,9,10]. Quantum dot (QD) nanocrystals have been proven advantageous in these approaches due to their highly efficient fluorescence, size-tunable Gaussian emission spectrum, excellent stability against photobleaching, large Stokes shift and low background signal [11,12,13]. However, given their photoluminescent character, portable QD readers are often sophisticated and relatively expensive; for example, they may require filters and/or lenses [14,15], or involve high-range mobile phones obviating the need for external optical filters [16]. Hence, generally, QD readers are not particularly accessible in relatively low-resource settings. In response, employing paper materials and a mid-range smartphone camera, we engineered a “do it yourself” QD reader.

## 2. Materials and Methods

Black matboard, cellophane paper, and electronic components were purchased in the local market (León, Guanajuato, Mexico). The laminated cards, nitrocellulose membrane, sample and absorbent pads for the production of the lateral flow strips (LFS) were purchased from Millipore (Billerica, MA, USA, https://www.merckmillipore.com). Streptavidin−quantum dot 655 (CdSe@ZnS) was from Life Technologies (Carlsbad, CA, USA, https://www.thermofisher.com). LFS were spotted with 2 µL of QDs at different concentrations, from 2.5 nM to 20 nM. After the spotting process, the LFS were dried at room temperature overnight to be then analyzed. A USB4000 UV–Vis (ultraviolet–visible) spectrometer (Ocean Optics, Inc., Largo, FL, USA, https://www.oceaninsight.com) was utilized to acquire the light-emitting diode (LED) emission spectrum. A Cytation 5 multimodal spectrometer (BioTek Instruments, Inc., Winooski, VT, USA, https://www.biotek.com) was employed to record the emission spectra of the QDs and nitrocellulose autofluorescence, as well as the absorbance spectra of the paper-based filters. Limit of detection (LOD) was estimated by interpolating the average of the intensity value of the blank sample plus 3 times its standard deviation within the respective calibration plot. Limit of quantification (LOQ) was estimated by interpolating the average of the intensity value of the blank sample plus 10 times its standard deviation within the respective calibration plot.

*Safety*. In order to avoid damage of the reader in long-term use due to possible LED overheating, it is recommended to use the reader less than 20 min per analysis. If the analysis requires more time, the device is recommended to be turned off for 5 min and the reader can be then utilized again. No heating effect was spotted on the analyzed sample during fluorescence interrogation. Moreover, all the internal edges of the case should be cautiously sealed (for instance, using a black tape) in order to avoid possible UV radiation exposure.

## 3. Results and Discussion

### 3.1. Design of the Paper-Based Quantum Dot (QD) Reader

**Figure 1:**
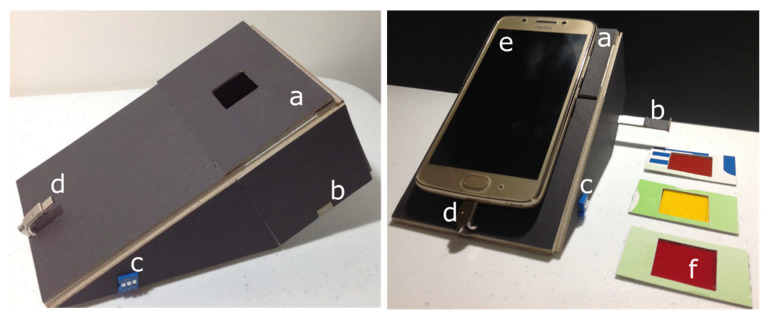
Pictures of the paper-based quantum dot (QD) reader. (**a**) External filter holder. (**b**) Tray to introduce the sample into the reader. (**c**) Illumination angle control. (**d**) Universal serial bus (USB) connector to power the excitation source. (**e**) Moto G5. (**f**) Paper-based filters.

Firstly, considering the dimensions, camera and universal serial bus (USB) specifications of the employed smartphone [Moto G5, see Table S1 in the Supplementary Materials, (SM)], we designed a foldable paper model of the reader case. Figures S1 and S2 (detailed in SI) detail the characteristics of such a papercraft, which was implemented using black matboard. The color of this type of paper was chosen to exhibit a low background when exposed to the excitation source, that is, a violet LED; thus potentially minimizing undesired noise during the imaging process. The papercraft includes paper based filters, an external filter holder, a tray to introduce the sample into the reader, an illumination angle control, as well as a USB connector to take advantage of the smartphone battery to power the excitation source via the on-the-go configuration, see Figure 1 and Figure S2 (detailed in SM). 

**Figure 2:**
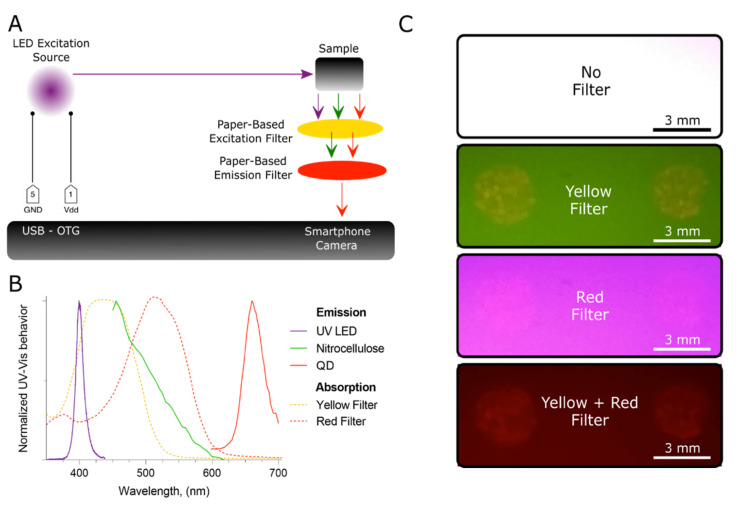
Optical components of the paper-based QD reader and their characterization via ultraviolet–visible (UV–Vis) spectroscopy. (**A**) Schematic representation of the optical path. (**B**) UV-Vis behavior of the optical components. (**C**) Images recorded with different filter configurations. The images were acquired through the smartphone camera.

With this hardware in hand, we proceeded to study the performance of the proposed QD reader. The optical path and the involved components are illustrated in Figure 2A. Two 5 mm round LEDs with emission wavelength centered at 400 nm, full width-half maximum (FWHM) around 14 nm, were employed to excite the QDs emitting around 655 nm. Figure S3A (depicted in SM) details the electronic circuit employed to power these light sources. The employed streptavidin-coated QDs are commercially available (Life Technologies, Carlsbad, CA, USA) and exhibit a rice-like shape with an average size around 14 nm [17], Figure 2B displays the respective emission spectrum. As model samples, LFS were manufactured following previous procedures [18]. To endow the imaging acquisition process with a relatively even illumination, we studied 3 illumination angles (180, 45 and 90°) and measured the coefficient of variation (CV) of the pixel intensities centered in the detection pads of the LFS, see Figure S3B,C (detailed in SM). Figure S3C (included in SM) demonstrates that the illumination angle at 45° resulted to show the lower CV, which accounted for 11.55%. Consequently, this illumination angle was chosen as optimal among the available illumination angles. 

### 3.2. Ultraviolet–Visible (UV–Vis) Characterization of the Paper-Based Filters

We also characterized the absorbance of the proposed paper-based filters using UV–Vis spectroscopy. Conveniently, the studied yellow cellophane exhibited an optical bandpass filter-like behavior with a central wavelength at c.a. 435 nm and a FWHM around 103 nm [19], see Figure 2B. Hence, this material was proposed as a paper-based excitation filter. The detection pad of the LFS is made of nitrocellulose. Using the proposed yellow filter during the imaging process, these detection pads were observed to display a strong green autofluorescence when excited with the employed light source emitting at 400 nm; see the corresponding emission spectrum in Figure 2B and a picture recorded under these conditions in Figure 2C. Hence, following color theory [19], we envisaged that a paper-based emission filter might be convenient to remove such a green noise. In this context, we explored the UV–Vis absorbance of a piece of red cellophane. This material also exhibited an optical bandpass filter-like behavior with a central wavelength at c.a. 510 nm and a FWHM around 110 nm, see Figure 2B. Eventually, using the proposed filters, we managed to acquire an image of the respective red emission of QDs spotted onto nitrocellulose at a relatively low concentration (2.5 nM), see Figure 2C.

### 3.3. Analytical Behavior of the Resulting QD Reader

**Figure 3:**
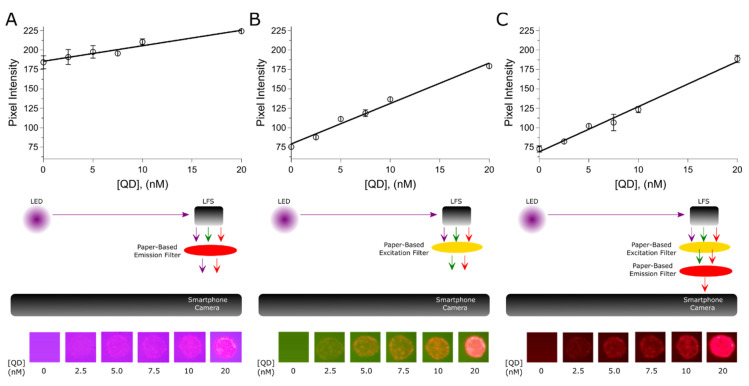
The resulting calibration plots under different paper-based filter configurations and the corresponding images. (**A**) Red filter. (**B**) Yellow filter. (**C**) Red + yellow filter. The error bars represent the standard deviation of two QD spots drop-casted onto lateral flow strips (LFS). The size of the spots is c.a. 3 mm.

Upon the aforementioned optical characterization, LFS were drop-casted with 2 µL of several QD concentrations (2.5, 5, 7.5, 10 and 20 nM) and we recorded the respective images using the paper-based QD reader under different filter configurations (red filter, yellow filter, yellow + red filter), see Figure 3. It is worth mentioning that hydrophobic walls created within paper via wax printing can enhance the variability of spots when drop-casted within wax-printed wells [20]. Although the pixel intensity of the acquired images can be directly analyzed in the smartphone using IJ_Mobile [21], we preferred to extract and handle these data by using MATLAB aiming at performing a controlled statistical analysis. Briefly, to extract pixel intensities, an image binarization process was performed using Otsu’s method [22], 0 values were considered background and 1 values were considered the QDs’ signal to build a binary mask. This binary mask was used to define regions of interests and extract the studied pixel intensities, Figure S4 (included in SM) shows an example of this process. By means of the resulting pixel intensities, we performed the respective calibration plots, see Figure 3. The value of the inverse of the slope of the resulting curves sheds light on the sensitivity of the respective configuration, whereas the resulting Y-intercept value offers information on the baseline. Generally, images recorded with the red filer configuration show a strong violet background, triggering a baseline accounting for c.a. 186 pixel intensity units at the blank signal. The sensitivity of this configuration accounts for 0.5 nM of QDs per pixel intensity units. As mentioned before, images captured with the yellow filter configuration show a green background. However, the corresponding baseline accounts for c.a. 79 pixel intensity units and the respective sensitivity is around 0.19 nM of QDs per pixel intensity units. As depicted in Figure 3C, the yellow + red filter configuration showed a relatively cleaner imaging process. The resultant baseline was around 69 pixel intensity units and the corresponding sensitivity accounted for 0.17 nM of QDs per pixel intensity units. Table 1 summarizes these analytical details.

Prompted by these results, we performed a comparative study by analyzing the same LFS using a commercially available equipment specially designed to measure QDs emitting at 655 nm onto LFS (ESEQuant LR3, QIAGEN, Hilden, Germany). Figure S5 (included in SM) displays the profiles resulting from the photoluminescent intensity of the QD spots onto the LFS analyzed by ESEQuant LR3. The average value of the profile corresponding to the analyzed QD spot was chosen as the analytical parameter to build the corresponding calibration plot (arbitrary units). However, the analyzed concentration range (2.5–20 nM) did not fit a linear response with an acceptable coefficient of determination (R^2^), which accounted for c.a. 0.9127, see Figure S6A (detailed in SM). Eventually, we sought a linear response within a QD concentration range from 2.5 to 10 nM, which offered an improved R^2^ (0.9772) and a sensitivity of 0.06 nM of QD per arbitrary unit, see Figure S6B (displayed in SI) and Table 1. Hence, in terms of the 1/slope value, ESEQuant LR3 was observed to be 2.75 times more sensitive than the paper-based reader incorporating the red + yellow filter configuration. In contrast, the paper-based reader offered a broader linear range in the explored concentrations as detailed in Table 1. LODs and LOQs were also estimated. As observed in Table 1, in terms of the LOD, ESEQuant LR3 was observed to be 3.02 times more sensitive than the paper-based reader incorporating the yellow filter configuration. ESEQuant LR3 was also observed to offer a LOQ 15 times lower than that of the paper-based reader incorporating the yellow filter configuration. Table 1 also highlights that the reader resulted to be more sensitive with the red + yellow filter configuration in terms of the 1/slope value, whereas the lowest LOD offered by the paper-based reader was achieved with the yellow filter configuration. Hence, the resulting paper-based bandpass filters were proven to be technically sound by evaluating these analytical parameters.

Importantly, the proposed paper-based QD reader can be considered a low-cost device, it is approximately 1877-fold cheaper than ESEQuant LR3 and does not require a high-range smartphone camera. Furthermore, this paper-based device obviates the usage of expensive filters or lenses. Table 2 highlights these competitive advantages. 

## 4. Conclusions

A cost-effective paper-based photoluminescent QD reader is reported. Black paperboard facilitated a lightweight and foldable case. Given the versatility offered by this material, the case can be redesigned easily to be compatible with other mobile phones. Moreover, cellophane paper was observed to behave as a simple optical bandpass filter leading to an advantageous device for quantitative interrogation of QD concentrations that are suitable for optical point-of-care biosensing [18]. Although the fabrication of this device may require previous skills in electronics and engineering, other types of readings such as optical density or chemiluminescence can be performed by properly adapting the design of the proposed reader. This reader is also amenable to the analysis of different disposable sensors based on fluorescence, including LFS, vertical flow, dip-stick and microfluidic paper-based analytical devices. 

## 5. Patents

Patent application under preparation.

## Figures and Tables

**Table 1:** Analytical performance of the studied QD readers.

Configuration	Linear Model	R^2^	1/slope	LOD ^1^ (nM)	LOQ ^2^ (nM)	Linear Range (nM)
Red filter	Y = 1.994*X + 185.6	0.9455	0.5014	11.856	42.201	2.5–20
Yellow filter	Y = 5.206*X + 79.12	0.9834	0.1921	0.918	4.744	2.5–20
Red + yellow filter	Y = 5.81*X + 69.06	0.9892	0.1721	2.773	7.778	2.5–20
ESEQuant	Y = 15.98*X − 3.454	0.9772	0.06256	0.303	0.314	2.5–10

^1^ Limit of detection. ^2^ Limit of quantification.

**Table 2:** Photoluminescence readers for point-of-care applications.

Reported Price (USD)	Filters/Lens	Smartphone	Reference
8450	Not specified	–	ESEQuant LR3
10 ^1^	No	High-range (iPhone SE or Nexus 5)	[16]
5 ^2^	Yes/Yes	High-range (iPhone 5s)	[23]
4.5 ^3^	Yes/No	Mid-range (Moto G5)	This work

^1^ Smartphone not included. ^2^ Filters, lens and smartphone not included. ^3^ Paper-based filters included, smartphone not included.

## Supplementary Materials

The following are available online at https://www.mdpi.com/2079-6374/10/6/60/s1: Figure S1. Foldable papercraft of the proposed QDs reader; Figure S2. A. Lateral view of the reader case. B. Top view of the reader case; Figure S3. A. Scheme of the employed electronic circuit; Figure S4. Image processing and pixel intensity estimation; Figure S5. Experimental evidence. Lateral flow strips with different QDs concentrations analyzed using ESEQuant LR3 (QIAGEN, Hilden, Germany); Figure S6. Calibration plots resulting from the calculation of the area below the curves representing the QDs intensities measured by ESEQuant LR3 and Table S1. Smartphone (Moto G5) specifications.
